# The Impact of Integrin-Mediated Matrix Adhesion on Cisplatin Resistance of W1 Ovarian Cancer Cells

**DOI:** 10.3390/biom9120788

**Published:** 2019-11-26

**Authors:** Kathleen Wantoch von Rekowski, Philipp König, Svenja Henze, Martin Schlesinger, Piotr Zawierucha, Radosław Januchowski, Gerd Bendas

**Affiliations:** 1 Department of Pharmacy, University of Bonn, 53121 Bonn, Germany; kathleen.wantoch@uni-bonn.de (K.W.v.R.); s6pikoen@uni-bonn.de (P.K.); svenja.henze@uni-bonn.de (S.H.); martin.schlesinger@uni-bonn.de (M.S.); 2 Department of Anatomy, Poznań University of Medical Sciences, 60-781 Poznań, Poland; pzawierucha@ump.edu.pl; 3 Department of Histology and Embryology, Poznań University of Medical Sciences, 60-781 Poznań, Poland; rjanuchowski@ump.edu.pl

**Keywords:** cell adhesion mediated drug resistance (CAM-DR), cisplatin, collagen, integrin, ovarian cancer, chemoresistance

## Abstract

Background: Tumor cell binding to the microenvironment is regarded as the onset of therapeutic resistance, referred to as cell adhesion mediated drug resistance (CAM-DR). Here we elucidate whether CAM-DR occurs in ovarian cancer cells and contributes to still-existing cisplatin resistance. Methods: Cultivation of W1 and cisplatin-resistant W1CR human ovarian cancer cells on collagen-type I (COL1) was followed by whole genome arrays, MTT assays focusing cisplatin cytotoxicity, and AAS detection of intracellular platinum levels. Expression of cisplatin transporters Ctr1 and MRP2 was analyzed. Mechanistic insight was provided by lentiviral β1-integrin (ITGB1) knockdown, or inhibition of integrin-linked kinase (ILK). Results: EC_50_ values of cisplatin cytotoxicity increased twofold when W1 and W1CR cells were cultivated on COL1, associated with significantly diminished intracellular platinum levels. Transporter deregulation could not be detected at mRNA levels but appears partially responsible at protein levels. The ITGB1 knockdown confirms that CAM-DR follows a COL1/ITGB1 signaling axis in W1 cells; thus, a blockade of ILK re-sensitized W1 cells on COL1 for cisplatin. In contrast, CAM-DR adds to cisplatin resistance in W1CR cells independent of ITGB1. Conclusions: CAM-DR appears relevant for ovarian cancer cells, adding to existing genetic resistance and thus emerges as a target for sensitization strategies.

## 1. Introduction

Chemoresistance, referred to as the loss of tumor sensitivity to the cytotoxic activity of antineoplastic drugs, remains the major restraint in the clinical treatment of cancer patients. Most resistance phenomena are based on an adaptive genetic reprogramming of the tumor cells upon cytotoxic therapy. The molecular resistance mechanisms are versatile, and the functional consequences at the cellular level range from mutations in the signaling pathways to circumvent apoptosis and to trigger proliferation, to changes in the intracellular processing of the drugs, most likely associated with an increased drug efflux by ATP-dependent (ABC)-transporters [1,2].

These clinical considerations are highly relevant for epithelial ovarian cancer, the second most common and the most lethal gynecological malignancy [3]. The reason for the high mortality is, on the one hand, related to the late diagnosis of ovarian cancer, since most patients are diagnosed at an advanced stage of the disease associated with a bad prognosis. On the other hand, resistance formation of the tumors against the chemotherapeutic treatment is a further important factor with a strong impact on overall survival. In principle, the majority of patients respond well to chemotherapy after a cytoreductive surgery, which encompasses platinum drugs and paclitaxel [4,5]. However, ovarian cancer has a high recurrence rate, which requires a continuation of the treatment, including liposomal doxorubicin, topotecan, or gemcitabine in the case of platinum insensitivity [4]. Treatment conduction often goes along with a resistance formation against this spectrum of cytotoxic drugs, which illustrates the versatility of resistance mechanisms.

Environmental mediated drug resistance has attracted much attention during the last years [6,7,8]. The role of the microenvironment, either represented by soluble factors, fibroblast or myeloid host cells, or more likely components of the extracellular matrix (ECM), such as collagen, laminin, or fibronectin as binding partners, has been recognized as important initial protection mechanism of tumor cells. Binding to ECM components, also referred to as cell adhesion-mediated drug resistance (CAM-DR), appears as a functional onset to escape cytotoxic stress rapidly at a non-genetic basis and, thus, as a premise for subsequent genetic adaptations. CAM-DR, which has initially been described in multiple myeloma and other malignancies of hematopoietic origin [9], also appears relevant for different solid tumor entities [10,11,12]. In a recent study, Zhu et al. investigated clinical specimens of ovarian cancer and correlated the expression of CAM-DR related markers, such as CD44, CD147, α5, or β1-integrin subunits with higher resistance and a worse prognosis [13].

This study gives a link to CAM-DR in the clinical setting of ovarian cancer. Nevertheless, the molecular mechanisms of CAM-DR remain elusive. Considering the key role of CAM-DR as an early form of resistance formation, an insight into the underlying molecular pathways could provide novel targets for early pharmacological interference with resistance formation.

Integrins, the heterodimeric adhesion that molecules possess, depending on their subunit composition, the capability to bind ECM components. Since integrin binding activity does not only regulate a physical cell embedding into an ECM, but also activates specific signaling pathways, integrins appear as relevant candidates for mediating CAM-DR. We have recently shown that the β1-integrin (ITGB1) plays a crucial role in breast cancer and melanoma cell lines, forming a resistant state against cytostatic drugs when cells bind to ECM components [14,15].

Although several studies illustrate a link between integrins and increased malignancy or resistance of ovarian cancer cells, a generalization of CAM-DR mediated mechanisms remain open. Tumor integrin β8 expression was shown to be an independent prognostic indicator of unfavorable outcomes of patients with serous ovarian cancer [16]. Wei et al. reported on the upregulation of integrin α6 subunit in resistant SKOV3 and A2780 ovarian cancer cells and correlated these findings with clinical samples of resistant tumor tissues [17]. Recently, Chang et al. demonstrated that the drug-resistant subline of SKOV3 cells exhibit a slug activated c-Met hyperactivation and associated this with integrin activities [18]. Related to matrix component effects, the upregulation of collagen triple helix repeat containing-1 by ovarian cancer cells was shown to trigger metastasis of ovarian cancer via an integrin β3/FAK signaling pathway [19].

Aiming to elucidate the relevance and underlying mechanisms of CAM-DR in ovarian cancer cells, the present study investigates the role of collagen binding of W1 ovarian cancer cells with respect to cisplatin sensitivity. To figure out whether CAM-DR is an additional independent process or an integrative part of an already existing cisplatin resistance, we also apply a cisplatin-resistant subtype of W1 cells (W1CR).

Here we provide evidence for a direct link between ITGB1-mediated matrix binding properties and CAM-DR in W1 and W1CR cells, resulting in significantly attenuated cisplatin sensitivity. Although CAM-DR is relevant in both cell lines in addition to the existing cisplatin resistance in W1CR cells, the β1-integrin signaling induces different signaling pathways in both cell lines. 

## 2. Materials and Methods

### 2.1. Cell Culture

The human ovarian cancer cell line W1 and the cisplatin resistant subtype W1CR have been described before [20]. The W1 cell line was established using ovarian cancer tissue received from an untreated patient. Cisplatin resistance of the W1CR subline was obtained by exposing W1 cells to cisplatin at incrementally increased concentrations [20]. W1 and W1CR cells were cultivated at 37 °C and 5% CO_2_ in RPMI 1640 medium containing 10% FCS and 1% penicillin. Cells were detached using a solution of EDTA (0.2 g/L EDTA × 4 Na) for 2 min at 37 °C. In order to ensure the resistance for a longer period, 1000 ng/mL cisplatin was added to the W1CR cell medium. All reagents were from PAN Biotech GmbH, Aidenbach, Germany. The maintenance of cisplatin resistance in W1CR cells, as well as the absence of mycoplasma in cell culture, was confirmed every second week.

Ninety-six-well plates were coated with collagen type 1 (COL1) (Corning, Thermo Fisher Scientific Inc., Waltham, USA) at a density of 10 μg/cm^2^, according to the manufacturer’s protocol. For Western blot experiments, collagen-coated cell flasks were used (Sarstedt AG & Co, Nümbrecht, Germany). As an inhibitor of ILK, cdp22 (Merck Chemicals GmbH, Darmstadt, Deutschland) was used at a concentration of 1 µM added to the cells 1.5 h prior to cytostatic treatment.

For the 3D cell culture model, 0.15 g of agarose (Carl Roth GmbH & Co. KG, Karlsruhe, Germany) was added to 10 mL of RPMI medium in an appropriate vessel and autoclaved for 20 min at 120 °C, 2 bar. Thereafter, the agarose was kept in a preheated water bath on a heating plate at about 60 °C under sterile conditions to avoid cooling down and solidification. Under sterile conditions using a manual precision dispenser, 50 mL of agarose suspension was distributed to each well of a 96-well sterile, black, flat bottomed plate with a lid (Greiner Bio-One GmbH, Frickenhausen, Germany). The agarose solidified within seconds to minutes. After cooling down to room temperature, the plates were packed in aluminum foil, in which they could be used for 10 d after production (hermetically sealed and stored at room temperature). For the following experiments, the cells were seeded as for MTT experiments as described below. 

### 2.2. β1-Integrin Knockdown of W1 and W1CR Cells

β1-integrin of W1 and W1CR cells was knocked down using viral transduction. First, cells were seeded in a 96-well plate at a density of 1000 cells per well and incubated overnight in RPMI medium containing additives. The next day, the medium was substituted by 100 µL per well of transduction medium (Polybrene^®^ 4 μg/mL in complete cell medium). Also, the viral particles (control shRNA lentiviral particles-A and ITGB1 shRNA (h) lentiviral particle: sc-35674-V; Santa Cruz Biotechnology, Heidelberg, Germany) were thawed and resuspended. In one well, 4 μL of integrin β1 shRNA (h) lentiviral particles were added to the medium, the same procedure was carried out for the control shRNA lentiviral particles in another well. One day later, the media was changed, and the still infectious medium containing lentiviral particles were used to transfect another well in the same manner. The cells were incubated in a medium containing 2.75 µg/mL puromycin (Carl Roth GmbH) to enable the selection of stable clones. The transfected cells were further cultured until they nearly reached confluence and then passaged for further experiments. Knockdown was confirmed by Western blot using a mouse anti-β1-integrin antibody [P2D5] (Santa Cruz Biotechnology). 

### 2.3. Cytotoxicity Assay

The cytotoxicity of cisplatin (Sigma-Aldrich Chemie GmbH, Steinheim, Germany) in W1 and W1CR cells and respective effects of the indicated inhibitor was determined by an MTT assay using 3-(4,5-dimethylthiazol-2-yl)-2,5-diphenyltetrazolium bromide (BioChemica, Applichem GmbH, Darmstadt, Germany) as described in [21]. Cells were seeded at a density of 5 × 10^3^ cells/well and 1 × 10^4^ in triplicates in 96-well plates, respectively (Sarstedt AG & Co) or collagen-coated plates, as described before. The next day, cells were supplemented with a dilution series of cisplatin (10^−3.3^ to 10^−7.5^ M). After 72 h of incubation, the MTT solution (20 µL, 5 mg/mL) was added into the wells for 1 h at 37 °C and 5% CO_2_. Subsequently, the supernatant was replaced, and formazan was solubilized in 200 µL DMSO. All plates were analyzed under a spectrophotometer at 570 nm, with background subtraction at 690 nm, using a plate reader (Thermomultiscan EX, Thermo, Schwerte, Germany). 

The alamarBlue^TM^ assay (Sigma-Aldrich Chemie GmbH), which was performed to also measure cytotoxicity in the 3D cell cultures, applied a resazurin solution. The cells were seeded in 2D and 3D, as indicated above. On the following day, 10 µL/well of cisplatin logarithmic concentration curve was applied to the cells; the total volume was also 100 µL per well. After 72 h incubation at 37 °C with 5% CO_2_, 20 µL of a resazurin solution of 0.15 mg/mL in wash buffer (0.5% BSA, 0.1% sodium azide, DPBS) was added to each well and incubated for 3 h, at 37 °C, with 5% CO_2_. Afterward the plates were analyzed using a FLUOstar^TM^ OPTIMA fluorescence scanner (BMG Labtech GmbH, Offenburg, Germany), with a 560 nm excitation and 590 nm emission filter.

### 2.4. Detection of Intracellular Platinum Concentration

The cisplatin uptake by both cell lines was analyzed by flameless atomic absorption spectrometry (fAAS). For this purpose, cells were treated using the following protocol. After a 72 h incubation period with a concentration of cisplatin (1.0 µM) in normal cell culture flasks or collagen-coated bottles (Sarstedt AG & Co), the medium was discarded and the cells were washed once in cold DPBS. After removing DPBS, cells were trypsinized for 2 min and resuspended in fresh medium and centrifuged at 1580× *g* and 4 °C for 4 min. The cell pellet was resuspended in 1 mL DPBS. In the next step, 20 μL were taken from this mixture and frozen at −20 °C until further analysis of the total protein concentration of the cells with a Pierce™ BCA Protein Assay Kit (Thermo Fisher Scientific Inc., GmbH, Darmstadt, Germany). The remaining suspension was centrifuged again, and the supernatant was removed. This washing step was repeated a second time. Finally, the cell pellets were stored at −20 °C until further processing.

Toward thawing each cell pellet, 50 µL of suprapur 65% nitric acid were added and lysed at 60 °C in a water bath for 1 h. The samples were diluted with 6.5% nitric acid and analyzed by fAAS using a modification of the procedure described [22]. An atomic absorption spectrometer (SpectrAA™ Zeeman 220; Varian, Darmstadt, Germany) was used. The temperature program comprised a pretreatment temperature of 1300 °C and an atomization temperature of 2700 °C. Platinum concentrations were related to the cell number (measured by Casy™ 1 cell counter, Schärfe System, Reutlingen, Germany).

### 2.5. Western Blot

Cell protein lysate was obtained using cell extraction buffer (Life Technologies, Carlsbad, CA, USA) followed by incubation for 30 min, at 4 °C, on a shaker. After centrifugation, the supernatant was collected and submitted to protein quantification by a BCA Protein Assay Kit. SDS-Page and Western blots were performed as described using stain-free gels [15]. Membranes were incubated with mouse anti-GAPDH, mouse anti-ILK [N1C1] (GeneTex, Irvine, USA), mouse anti-β-actin, mouse anti-β1-integrin P5D2, rabbit anti-CTR1 [FL190], goat anti-MRP2 [H17] (Santa Cruz Biotechnology), as well as goat anti-rabbit, donkey anti-goat and anti-mouse IgG kappa binding protein IgG HRP-conjugated (Santa Cruz Biotechnology) diluted in 1% BSA solution. Western blots were quantified via chemiluminescence using a Clarity Western ECL substrate chemiluminescence kit (BioRad Laboratories GmbH, Munich, Germany). Besides the loading control GAPDH, we also used stainfree total protein normalization. Membranes were photographed and analyzed using a ChemiDoc XRS+ imaging acquiring system (BioRad) and Image Lab software v. 6.0 (BioRad).

### 2.6. Glutathione Fluorescent A

A glutathione fluorescent detection kit (Invitrogen GmbH, Karlsruhe, Germany) was performed to analyze the amount of free glutathione (GSH) in W1 and W1CR cells. For this, cell lysates were made as already explained above with different treatments. A Pierce™ BCA protein assay kit was used to quantify total protein. The assay was performed according to the manufacturer’s instructions. After incubation at room temperature, the 96-well plate was measured in a FLUOstar Omega Fluorescence (BMG Labtech) at 510 nm with an excitation of 390 nm.

### 2.7. Microarray

The samples were hybridized on Affymetrix GeneChip human genome U219 microarrays, together with control cRNA and oligo B2. Hybridization was conducted at 45 °C for 16 h, using an AccuBlock™ Digital dry bath (Labnet International, Inc., New York, NY, USA) hybridization oven. Further, the microarrays were washed and stained according to the manufacturer’s protocol using an Affymetrix GeneAtlas™ Fluidics Station (Affymetrix, Santa Clara, CA, USA). In the final step, all microarrays were scanned using an Affymetrix GeneAtlas™ imaging station (Affymetrix, Santa Clara, CA, USA). The scans of the microarrays were saved as *.CEL files on local storage.

All microarray results are available in GEO database under ID GSE140996.

In order to perform higher levels of analysis, the *.CEL files were imported into Transcriptome Analysis Software (TAC version 4.0.1.36, Waltham, MA, USA). TAC, apart from visualization and a QC check of the data, allows the performance of normalization, background correction, and the creation of differential expressed gene (DEG) tables of user-defined comparisons. Each table of interest was exported to an .xlsx file for further analysis using R (version 3.6.1) and RStudio (version 1.1.463). In the next step, each .xlsx file was imported into the R ecosystem, where the number of DEG were limited to a threshold (above or under given fold change). From the limited list of DEGs, Affymetrix target IDs were extracted and imported to the web-based DAVID service (https://david.ncifcrf.gov) to conduct functional annotation to three gene ontology (GO) group families: biological process (BP), cellular component (CC), and molecular function (MF). Results of annotation were saved as a .txt file and imported to the R ecosystem. Subsequently, only statistical significantly enriched GO groups (*p* < 0.05) and group terms containing phrases “apoptotic”, “MAPK”, “ECM”, and “collagen” were further analyzed. All operations on data were made by following packages: dplyr (table manipulation, version 0.8.3), ggplot2 and GOplot (data visualization; version 3.2.1 and 1.0.2, respectively), and readxl (import MS Excel files, version 1.3.1).

### 2.8. Statistics

Cytotoxicity data of the sigmoidal dose–response curves were evaluated by a nonlinear regression using the four-parameter logistic equation with variable hill slope to acquire sigmoidal dose–response curves and to determine the EC_50_ at the curves’ inflexion point (GraphPad 6.0 Software, San Diego, CA, USA). The ratio of the EC_50_ of W1CR and the EC_50_ of W1 cells was calculated and referred to as to the resistance factor (RF). Moreover, statistical analysis was performed using one-way ANOVA following Tukey’s multiple comparison test (*p < 0.05; **p < 0.01; ***p < 0.001).

## 3. Results

### 3.1. Insight into Cisplatin Resistance of W1CR Ovarian Cancer Cells

**Figure 1:**
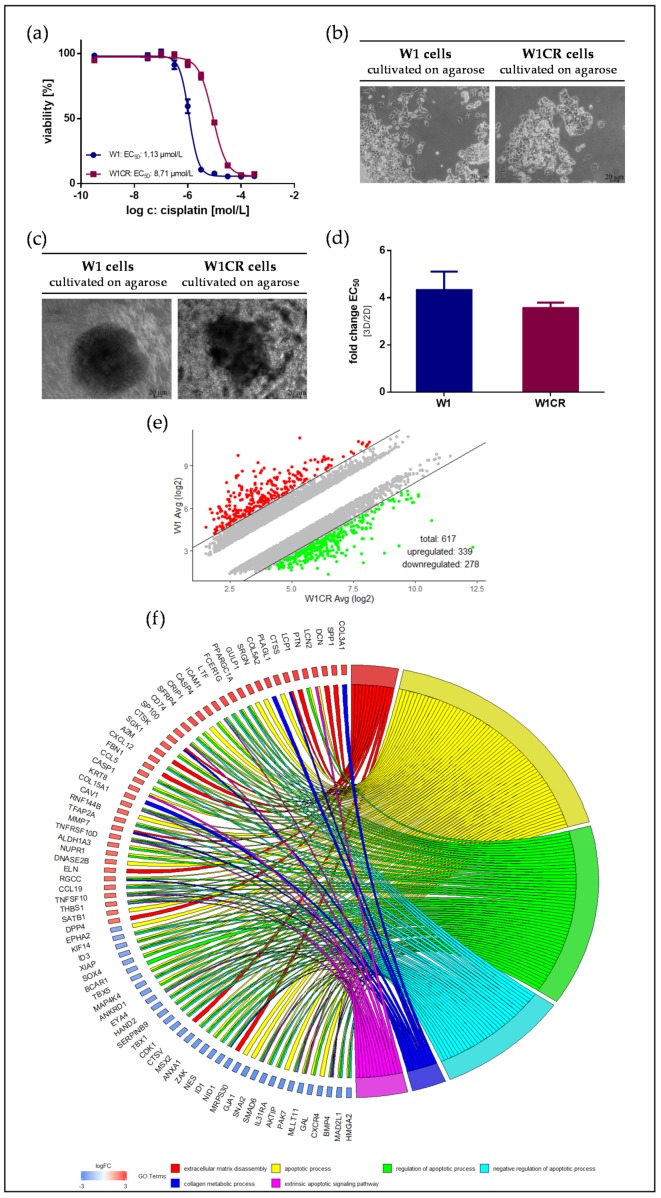
Characteristics of W1 and W1CR ovarian cancer cells with respect to cisplatin cytotoxicity and cell growth: (**a**) Representative data set of an MTT assay of W1 and W1CR cells (5000 cells) treated with cisplatin displays the higher resistance of W1CR cells. (**b**) Microscopic images of the growth characteristics of W1 cells (left) and W1CR cells (right) on agarose-coated surfaces. W1 cells tend to form closer cell aggregates. (**c**) 3D-spheroid formation of W1 cells (left) and W1CR cells (right) on agarose and (**d**) comparison of the impact of 2D and 3D cell cultivation on EC_50_ values of cisplatin cytotoxicity. Therefore, the ratio of EC_50_ values for 3D and 2D (detected by resazurin assay) was calculated and illustrated. All data are means of at least *n* = 3 (±SEM). (**e**) Scatter plot of upregulated and downregulated genes (fold change >5 / <−5) in W1CR compared to W1 cells in an untreated state. An exact list of the deregulated genes is given as an excel file in the Supplementary Materials (dereg_genes_W1CRvsW1.xlxs) (**f**) Chord plot representing an association between deregulated genes of W1CR cells and impacted GO terms. Additionally, a heatmap was performed and is shown in the Supplementary Materials (HM_related_to_Fig1f_W1CR_vs_W1.tiff).

The cisplatin-resistant subline W1CR displays a significantly reduced sensitivity to cisplatin cytotoxicity, indicated by a roughly eightfold higher EC_50_ value compared to W1 cells, obtained by MTT assays (Figure 1a, Table S1). However, resistance formation is not associated with evident morphological changes, both W1 and W1CR cells appear in a comparable growth characteristic at similar cell densities. Merely W1 cells tend slightly to grow in agglomerates, while W1CR cells are distributed in smaller cell clusters (Figure 1b). This is also reflected in a higher tendency of W1, compared to W1CR cells, to form spheroids in a 3D agarose culture (Figure 1c). To check the cytotoxicity of cisplatin in the 3D spheroids of both cell types, we had to apply the Alamar Blue^TM^ / resazurin assay. The EC_50_ values obtained by this assay for both the 2D and 3D cultivation of the cells (Figure S1) were compared and expressed as fold-change (ratio EC_50_-3D/EC_50_-2D) in Figure 1d. This ratio is slightly higher in W1 cells, reflecting the denser spheroid structure in W1 cells, and in consequence, the limited drug diffusion. 

The W1 cell line and sublines resistant against different cytotoxic drugs have undergone a genetic screening in recent studies [23,24,25]; nevertheless, the molecular mechanisms for cisplatin resistance remain to be elucidated. An actual comparison of W1 and W1CR cells at the mRNA level in a non-treated state by microarray analysis of the whole transcriptome displayed an increased expression of 339 genes and decreased expression of 278 genes in W1CR cells in comparison to W1 cells (Figure 1e). An exact list of the deregulated genes is given as an excel file in the Supplementary Materials (dereg_genes_W1CRvsW1.xlxs). To get a functional insight into the genetic regulation associated with resistance, we performed a DAVID analysis to extract statistically significant GO terms in the W1CR/W1 cell line comparison. Our result showed enriched GO terms in the biological process (BP) family with phrases associated with apoptosis and ECM/collagen. Additionally, apoptosis gene-related GO groups were balanced in expression level between W1 and W1CR cells, while genes from ECM/collagen-related GO groups were upregulated in W1CR, apart from *NID1* and *CTSV* genes (Figure 1f). Additionally, a heatmap was performed and is shown in the Supplementary Materials (HM_related_to_Fig1f_W1CR_vs_W1.tiff).

**Figure 2:**
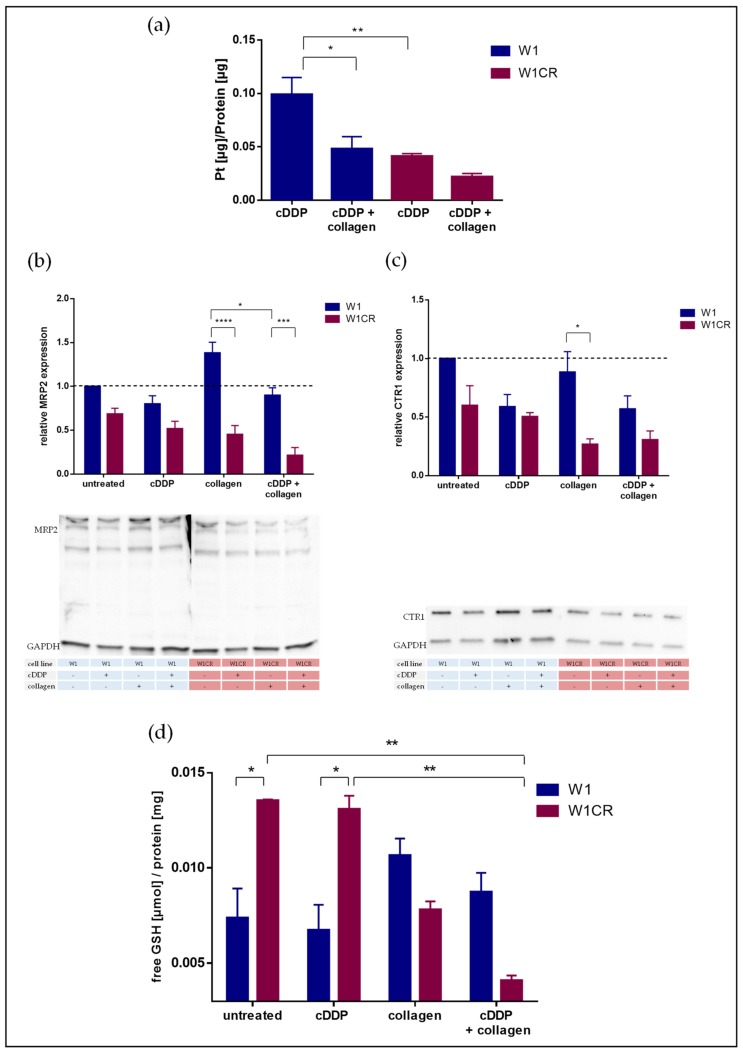
(**a**) Intracellular platinum concentrations of W1 and W1CR cells treated with 1 µM cisplatin analyzed by fAAS and the impact of cell binding to a COL1 matrix. (**b**,**c**) Western blot data of W1 and W1CR cells of the expression of the ATP-dependent (ABC)-transporter MRP2 (**b**) and the copper/platinum uptake transporter Ctr1 (**c**) and the impact of cell treatment with 1 µM cisplatin, cell cultivation on COL1, or both. (**d**) Investigation of the concentration of free glutathione (GSH) in both cell lines, untreated as well as treated with 1 µM cisplatin and/or collagen. Data are means of at least *n* = 3, asterisks indicate statistical significance: * *p* < 0.05; ** *p* < 0.01; *** *p* < 0.001; **** *p* < 0.0001.

Genetic deregulation of cellular transporters could not be detected here and in our recent studies [25]. Nevertheless, the intracellular platinum content was evaluated by fAAS to check whether resistance of W1CR cells is induced or associated by diminished cellular accumulation of the drug. Our data indicate that the platinum level in the W1CR cells is, by about 30%, lower compared to the identically treated W1 cells (Figure 2a). This might refer to the activity of cellular transporters. On the one hand, a downregulation of the copper transporter Ctr1 has been described to contribute to attenuated platinum levels. On the other hand, MRP2 is a known efflux transporter for cisplatin with respect to ovarian cancer resistance [26,27]. Therefore, we analyzed MRP2 and Ctr1 expression levels by Western blot (Figure 2b,c). It is evident that MRP2 is not significantly deregulated, actually expressed at a lower level in the W1CR cells, and surprisingly even lower expressed when cells were treated with cisplatin (Figure 2b). Since glutathione is known to serve as a cofactor in facilitating MRP2-mediated cisplatin efflux from the cells [28], we also checked the free glutathione (GSH) status of the cells. Notably, the data (Figure 2d) illustrate that W1CR cells, untreated or treated with cisplatin, have a significantly more than twofold higher concentration of GSH than W1 cells. Consequently, despite the lower expression of MRP2 in the W1CR cells, the higher GSH level could be an indicator for higher efflux capacities, explaining the lower cisplatin concentrations in W1CR cells (Figure 2a). 

Furthermore, the expression profile of the Ctr1 uptake transporter for cisplatin goes in line with the resistance behavior of the cells. The uptake transporter is much lower expressed in W1CR than in W1 cells (Figure 2c), which also explains the intracellular platinum data given above. Cisplatin treatment further downregulates the Ctr1 levels as a kind of cellular counter-regulation. 

The W1CR cells displayed certain genetic deregulation of ECM-associated genes, i.e., COL3A1 and COL5A2 (Figure 1f), which is in agreement with recent data of the other resistant subtypes of the W1 cell lines, such as W1 cells resistant to topotecan or paclitaxel [29]. Therefore, we aimed to elucidate the impact of those ECM substrates on cisplatin resistance of W1 and W1CR cells and checked whether cell cultivation at a matrix formed by collagen type I (COL1) affects platinum processing. Notably, in presence of COL1, the intracellular platinum concentrations in both cell lines are further reduced (Figure 2a). Interestingly, COL1 treatment does not affect the MRP2 expression nor the level of free GSH to facilitate cisplatin efflux in W1CR cells. However, the further downregulation of Ctr1 upon COL1 binding of W1CR (Figure 2c) can contribute to the lower intracellular platinum levels in the W1CR cells treated with COL1. 

Nevertheless, it remains questionable whether matrix binding of the actual W1 and W1CR cells additionally induces cisplatin resistance or is even responsible for the lower sensitivity to cisplatin in W1CR cells.

### 3.2. The Impact of Cell Binding to COL1 on Cisplatin Resistance of W1 and W1CR Cells

**Figure 3:**
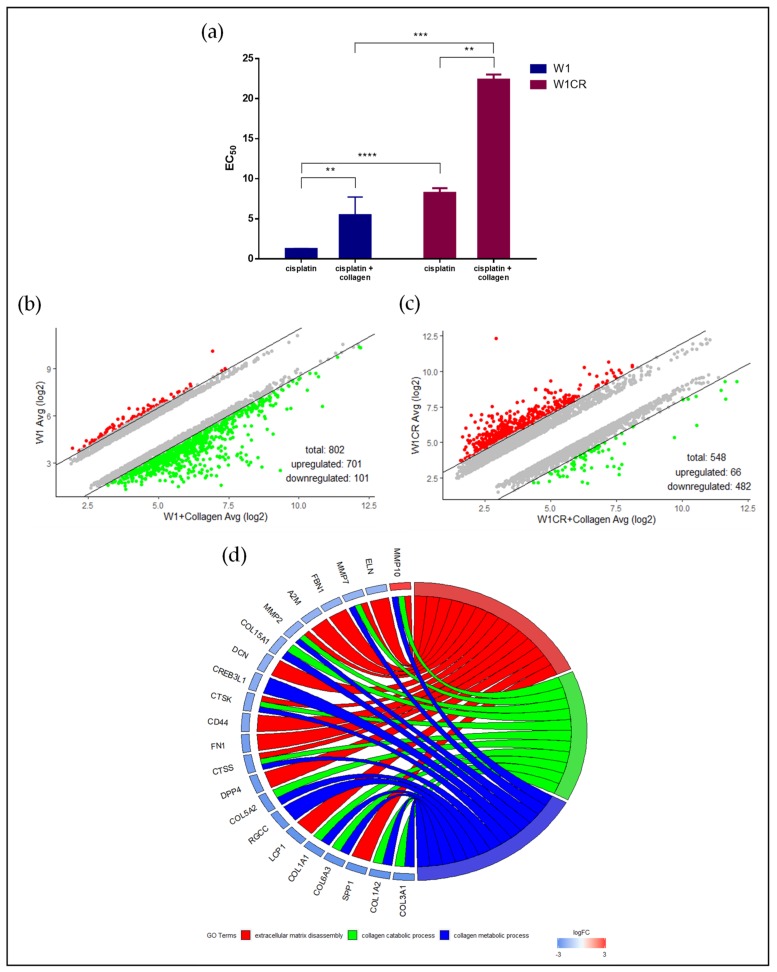
Cell adhesion mediated drug resistance (CAM-DR) in W1 and W1CR cells induced by COL1 binding: (**a**) Increase of EC_50_ values of cisplatin cytotoxicity in W1 and W1CR cells when cultivated on COL1 surfaces. Data are means of at least *n* = 3 (±SEM), asterisks indicate statistical significance: ** *p* < 0.01; *** *p* < 0.001; ****p < 0.0001. (**b**,**c**) Scatter plots of upregulated and downregulated genes (fold change >3 / <−3) upon cell cultivation on a COL1 matrix in (**b**) W1 cells and (**c**) W1CR cells, (**d**) Chord plot representing association between genes deregulated in W1CR cells upon cultivation on COL1 and gene ontology (GO) term analysis.

In order to investigate whether matrix binding affects the sensitivity of both cell lines to cisplatin, cells were cultivated on COL1-covered surfaces, which has been shown recently to induce a remarkable loss of sensitivity, e.g., breast cancer cells to different cytotoxic agents [15]. It becomes clear that both cell lines respond to COL1 binding by an evident increase in EC_50_ values, confirming the relevance of ECM substrates for CAM-DR formation in this scenario (Figure 3a). These findings are in agreement with the diminished intracellular platinum levels in the cells upon COL1 treatment, shown in Figure 2a.

The significant increase in W1CR resistance induced by COL1 points to the fact that CAM-DR is obviously an independent process contributing to the already existing cisplatin resistance of W1CR, but is not the basis for the “intrinsic” resistance. To further focus on the interference of cellular reactions to COL1 treatment and existing cisplatin resistance in W1CR cells, we again checked the mRNA deregulation at the genome level. First, we compared changes in gene expression of W1 cells incubated on COL1 coated surface and observed 701 genes upregulated and 101 genes downregulated after COL1 incubation (Figure 3b, supplementary list of deregulated genes W1 collagen vs. W1). Notably, the number of upregulated genes by COL1 treatment is crucially lower in W1CR cells (66 genes) than in W1 cells, but 482 genes were downregulated after incubation with COL1 (Figure 3c, supplementary list of deregulated genes W1CR collagen vs. W1CR). Interestingly, 136 genes of this downregulated populations belong to the upregulated genes of the “resistance signature” W1CR vs. W1, shown in Figure 1e. 

Next, we performed a DAVID analysis to extract statistically significant GO terms in a W1-COL/W1 and W1CR-COL/W1CR cell line comparison. We did not observe any enriched GO terms in the W1/W1-COL cell lines. In contrast, in the W1CR-COL/W1CR data set, all genes of the ECM/collagen GO enrichment data were evidently downregulated with exception of *mmp10* (Figure 3d). Alternatively, the deregulated pathways are illustrated in a heatmap format in Supplementary Materials (HM_related_to_Fig3d_W1CRCollagen_vs_W1CR).

### 3.3. The Role of β1-Integrins in COL1-Induced Cisplatin Resistance of W1 and W1CR Cells

**Figure 4:**
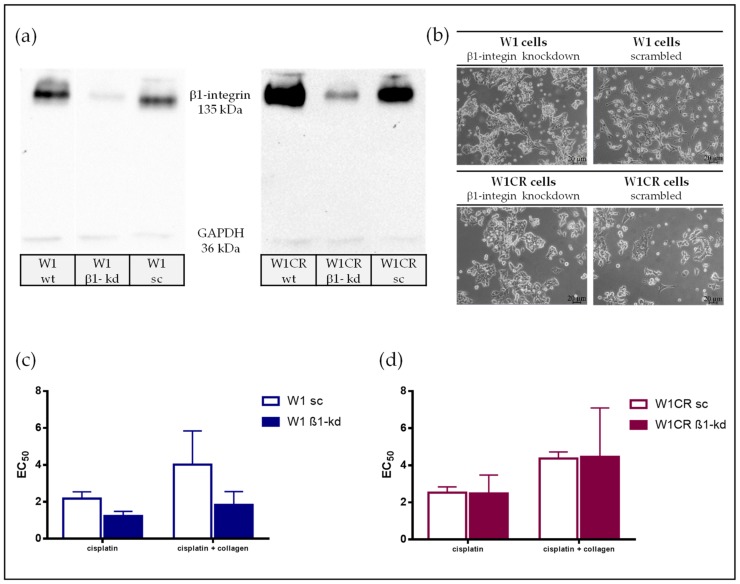
The impact of ITGB1 on CAM-DR in W1 and W1CR cells. (**a**) Representative Western blot data of ITGB1 knockdown in W1 and W1CR cells, compared to the indicated wild type and non-targeted scrambled knockdown cells. (**b**) Microscopic images of cultivation of W1-β1kd and W1CR-β1kd cells in a normal plastic flask compared to the respective wt cells indicate that ITGB1 kd induces a loss in surface contact formation of the cells. (**c**) ITGB1 knockdown in W1 cells clearly affects the cisplatin sensitivity resulting in lower EC_50_ values and a non-responding to COL1 binding induced CAM-DR. (**d**) ITGB1 knockdown in W1CR cells has no impact on a higher sensitivity to cisplatin. Data are means of at least *n* = 3 (±SEM).

Integrins appear as the most probable candidates to mediate COL1 binding, known to transfer ECM binding events into intracellular outside-in signaling, which can foster cell proliferation and survival. Since all COL1 binding integrins possess a β1 subunit, an interference with ITGB1 function should strongly affect resistance formation by COL1 binding in both cells. Aiming to elucidate this, we performed a lentiviral ITGB1 knockdown approach in W1 and W1CR cells. Both knockdown cells, W1-β1kd and W1CR-β1kd, display a compelling downregulation of ITGB1, compared to the corresponding wild type and scrambled control cells (Figure 4a). The absence of ITGB1 affects the contact formation of the cells with the cultivation surfaces, since both ITGB1 kd cell types tend to form closer cell agglomerates and avoid a spreading at the surfaces (Figure 4b).

Notably, the impact of ITGB1 knockdown on cisplatin sensitivity appears differently in both cell types. W1-β1kd cells become more sensitive to cisplatin when compared to the scrambled control. Remarkably, they do not respond to COL1 binding by higher EC_50_ values, which impressively confirm the ITGB1/COL1 axis (Figure 4c). 

In strict contrast, ITGB1 kd does not affect the cisplatin sensitivity in the W1CR cell line. Although one has to consider that the W1CR scrambled controls are not evidently more resistant than the W1 scrambled cells, probably as an indication for cell stress upon transfection, W1CR-β1kd cells have similar EC_50_ values as the scrambled control cells both in absence and presence of COL1 (Figure 4d). 

These data indicate that ITGB1-driven CAM-DR is a clear factor for cisplatin resistance in W1 cells when they are in contact with COL1. Interestingly, W1CR cells also respond to COL1 in the way of CAM-DR, additionally to the intrinsic cisplatin resistance. However, the signaling pathways appear, at least in part, different to W1 cells and independently from ITGB1.

### 3.4. The Impact of Blocking ILK Signaling in W1 and W1CR cells on Cisplatin Sensitivity

To investigate whether the diverse dependency of CAM-DR in W1 and W1CR cells from ITGB1 is manifested in intracellular integrin signaling, we focused on integrin linked kinase (ILK). ILK is a central signaling component of the integrin pathways, which impacts AKT and GSK3β by phosphorylation. 

**Figure 5:**
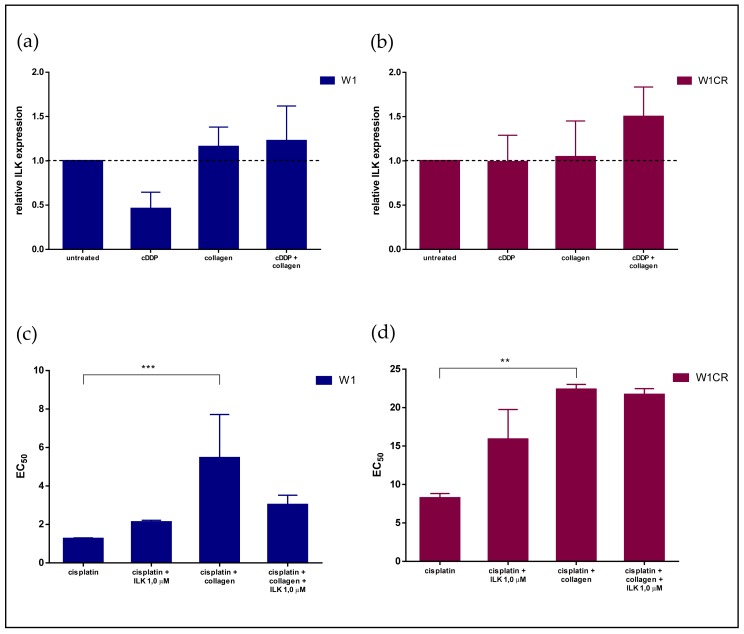
The impact of ILK on resistance formation in W1 and W1CR cells as a potential target for sensitization: (**a**,**b**) The expression of ILK in W1 (**a**) and W1CR (**b**) cells upon the indicated treatment regimens was detected by Western blot. Data indicate that W1 cells react to COL1 by higher ILK expression, which can obviously counterbalance the cytotoxic stress of cisplatin. The W1CR cells only marginally react to COL1 by deregulated ILK, but the combination of cisplatin with COL1 is indicated by a massive upregulation. (**c**,**d**) Blocking ILK by the small molecule inhibitor cdp22 at 1 µM concentration in W1 cells (**c**) clearly reversed the resistance effects of COL1, indicated by the EC_50_ values of cisplatin for the respective treatment regimes; while for the W1CR cells (**d**), no sensitivity is detectable. Data are means of at least *n* = 3 (±SEM), asterisks indicate statistical significance: ** *p* < 0.01; *** *p* < 0.001.

First, we compared the ILK expression in W1 and W1CR cells and the impact of cisplatin and COL1 by Western blot. While cisplatin treatment of W1 cells reduces the ILK expression, probably as a matter of apoptotic stress, COL1 induces an upregulation, which is also detectable in the combined treatment of COL1 and cisplatin. This appears indicative for the ITGB1/COL1 axis triggering the CAM-DR process (Figure 5a). The ILK expression in W1-β1kd cells matches with these findings; the kd cells display similar behavior unless the cells do not react to COL1 by an ILK upregulation, emphasizing the importance of the ITGB1 activation axis in presence of COL1 (Figure S2). 

In contrast, W1CR cells do not reduce ILK expression upon cisplatin treatment (Figure 5b), but COL1 does not induce ILK, staying at a similar level. However, the combination of cisplatin with COL1 led to a massive upregulation of ILK expression (Figure 5b).

Based on these findings, we investigated whether a functional blockade of ILK impacts cell sensitivity. Therefore, we preincubated the cells with the ILK inhibitor cdp22 before cisplatin treatment. At the concentration of 1.0 µM used, the inhibitor does not possess any intrinsic effects on cell survival (Figure S3). Interestingly, the increased EC_50_ values of cisplatin in the COL1 treated W1 cells were clearly reversed by blocking ILK (Figure 5c). This confirms the integrin dependency of the COL1 mediated cisplatin resistance in the W1 cells.

In contrast, W1CR cells do not react to ILK inhibition. The inhibitor seemingly increases the EC_50_ value of cisplatin, and more importantly, displays no interference with the COL1-induced resistance formation (Figure 5d). These data clearly confirm that the CAM-DR follows other pathways in the W1CR cells than the ITGB1 route relevant for W1 wild type cells. 

## 4. Discussion

Resistance formation of tumors against antineoplastic drugs remains the major obstacle in the clinical treatment of cancer patients. In terms of the environmentally-mediated communication of tumor cells, CAM-DR is meanwhile considered as an important initial trigger mechanism to escape apoptosis by a rapid, non-genetically based adaptation [30]. However, the underlying mechanisms of CAM-DR and the fact of whether and how it interferes with other types of resistance remain to be elucidated.

Here we demonstrate that W1 ovarian cancer cells binding to a COL1 matrix evidently protect them against cisplatin cytotoxicity. Although these data do not claim a generalization with respect to the in vitro background and the treatment with only one cytotoxic drug, they provide arguments for the process and relevance of CAM-DR in ovarian cancer cells. Furthermore, CAM-DR emerges as an independent process adding to the resistance phenomena already developed in ovarian cancer cells, which has not been shown before. Although the resistance rate, i.e., the shift to higher EC_50_ values of cytostatic drugs by matrix binding of cells is expected to be moderate or even low in terms of the non-genetic nature [14,15], the duplication of cisplatin EC_50_ values in both W1 and W1CR cells shown here, is remarkable. However, although these findings emphasize CAM-DR in its relevance, our present data have to be discussed and interpreted cautiously, for other ovarian cancer cells, also with respect to other types of ECM matrices, other environmental components, or antineoplastic drugs.

Concerning the mechanistic background of CAM-DR, although the binding to COL1 induces a comparable loss in sensitivity in both W1 and W1CR cells by doubling the EC_50_ values, the underlying mechanisms appear to be diverse, following different routes in both cells. In W1 cells, a clear axis emerges covering COL1 binding by ITGB1, thus inducing an intracellular integrin signaling that finally leads to reduced sensitivity. We cannot presently provide details of these intracellular processes inducing reduced apoptosis. Our gene array data refer to a massive upregulation in the number of genes upon COL1 binding, but did not reveal striking functional pathway deregulation. However, at a functional level, the COL1/ITGB1 axis is clearly confirmed by the ITGB1 knockdown approach in W1 cells. The W1-β1kd cells are more sensitive to cisplatin and still did not respond to COL1 binding by resistance formation. ILK was selected as a central signaling component of integrins associated with various cellular processes, such as proliferation, survival, adhesion, and migration [31]. ILK expression is clearly upregulated in W1 cells upon COL1 binding, but more impressively, ILK inhibition using a non-toxic concentration of the inhibitor cdp22 significantly sensitized the W1 cells for cisplatin cytotoxicity. This is comparable to recent studies in other tumor entities showing an interference with integrin signaling as promising targets for sensitization against CAM-DR [14]. 

In contrast, W1CR cells did not display an ITGB1 dependency in their COL1-mediated resistance, neither in the ITGB1 knockdown approach, nor in the ILK involvement. Obviously, other molecules appear responsible for mediating the COL1 binding and transfering this binding into an anti-apoptotic signaling pathway. Discoidin domain receptor 1 (DDR1) appears a potential candidate for collagen binding, which has recently been associated with malignancy, metastasis, and resistance in other tumor entities, such as breast cancer [32], gastric cancer [33], or pancreatic ductal adenocarcinomas [34]. Since DDR1 was shown to induce cell signaling, e.g., via the IGF axis in breast cancer [35], the insensitivity of W1CR cells to ILK inhibition could thus be explained. Although a recent study reported on an inverse relation of DDR1 expression and cisplatin sensitivity in ovarian cancer [36], a potential role of DDR1 in CAM-DR of W1CR cells remains a matter of further investigations.

Notably, the COL1 induced CAM-DR adds as an independent process to the pre-existing cisplatin resistance in W1CR cells, which is a novel and unexpected finding improving our understanding. The gene array data of the W1CR cells in the non-treated status display a certain upregulation of ECM-associated genes, such as COL3A1, COL5A2, and others (Figure 1f), which indicates a probable role of matrix formation in the intrinsic resistance. However, cell cultivation on COL1 appears to induce a genetic counter-regulation, which is evident by the massive downregulation in the number of genes (Figure 3d) and in the functional analysis of the ECM-associated pathways (Figure 3e). However, this could refer to a matrix-associated intrinsic resistance process at the genome level, which is assisted, and not antagonized by a COL1 induced, still not-known rapid adaptation via signaling pathways. 

Whether this pathway is functionally related in cisplatin transport, reflected in the intracellular platinum levels, remains partly open. The lower platinum levels in W1CR than in W1 cells are an obvious cause for lower cytotoxicity, which were further attenuated by COL1. The significantly higher GSH levels in the W1CR than in W1 cells undoubtedly provide a potential explanation, since GSH has been considered as an essential cofactor facilitating MRP2-mediated drug export. Although the functional details of GSH activity in cisplatin resistance have not fully been elucidated and remain partly controversial, as critically reviewed in [28], a potential role of GSH as cytoprotector against oxidative stress can explain higher resistance against cytotoxicity. In a recent study, the role of glutathione transferase 1-P-1 for cisplatin resistance has been addressed. It was shown that overexpression of this enzyme, leading to a platinum deactivation by protein subunit crosslinking, finally attenuates c-Jun N-terminal kinase (JNK) apoptosis signaling [37]. This contributes to the understanding of the tight relation between GSH overexpression and processing and cisplatin resistance. For our approach, it remains a matter of further investigations to decipher the interdependence of transporter activity and matrix binding.

In summary, the present findings emphasize matrix-assisted resistance formation as a highly relevant process worth being further addressed to find novel targets for clinical sensitization strategies in cancer patient treatments. 

## 5. Conclusions

Our data provide evidence for the relevance of CAM-DR in cisplatin treatment of W1 ovarian cancer cells, both for therapeutically naïve ones or, as a novelty, for those cells that have already developed a genetic resistance mechanism against this antineoplastic drug. Further insights based on these findings are needed to decipher the peculiarities of the underlying molecular mechanisms of CAM-DR in both cell types to generalize the importance of CAM-DR for ovarian cancer cells and for the treatment with other cytotoxic drugs as well. However, the interference with the matrix binding of ovarian tumor cells appears as an attractive target for sensitization strategies.

## Supplementary Materials

The following are available online at https://www.mdpi.com/2218-273X/9/12/788/s1: *dereg_genes_W1CRvsW1.xlxs*: Complete list of deregulated genes in W1CR cells compared to W1 cells, threshold level <-3/>3. *HM_related_to_Fig1f_W1CR_vs_W1.tiff*: heatmap for functional analysis of deregulated genes in W1CR cells versus W1 cells; *dereg_genes_W1collagen_vs_W1:* complete list of deregulated genes in W1 collagen treated cells vs. W1 cells; *dereg_genes_W1CRcollagen_vs_W1CR:* complete list of deregulated genes in W1CR collagen treated cells vs. W1CR cells; *HM_related_to_Fig3d_W1CRCollagen_vs_W1CR*: heatmap for functional analysis of deregulated genes in W1CR collagen treated cells versus untreated W1CR cells, threshold level <-3/>3. **Figure S1**: Characteristics of W1 and W1CR cells with respect to cisplatin cytotoxicity in a 2D vs. 3D model: (left) A representative data set of an alamarBlue^TM^ assay of W1 and (right) W1CR cells (10,000 cells) treated with cisplatin. For the 3D model the cells grew on agarose coated plates. This data is an example of at least *n* = 3. **Figure S2**: ILK expression profile in W1β1 kd cells. **Figure S3**: Concentration dependency of cell toxicity of the ILK inhibitor cdp22 in W1 and W1CR cells. The ILK inhibitor has no intrinsic cytotoxicity at the concentration used for interference with cell signaling. **Table S1**: EC_50_ values of cisplatin cytotoxicity in W1 and W1CR cells and the impact of COL1 binding.
