# Supplementary material for: The Impact of Integrin-Mediated Matrix Adhesion on Cisplatin Resistance of W1 Ovarian Cancer Cells
Source: Biomolecules. 2019 Nov 26;9(12):788. doi: 10.3390/biom9120788 (PMC6995566; doi:10.3390/biom9120788)
Supplement: Supplementary file 1 [file biomolecules-09-00788-s001.zip › biomolecules-644067-supplementary.pdf]

## Supplementary Materials

### Figures:

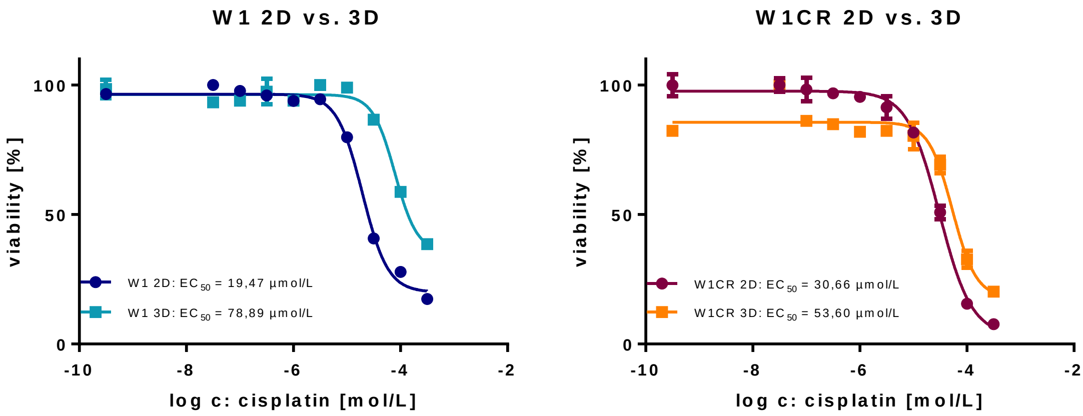


**Figure S1:** Characteristics of W1 and W1CR ovarian cancer cells with respect to cisplatin cytotoxicity in a 2D vs. 3D model: (left) A representative data set of an alamarBlue™ assay of W1 and (right) W1CR cells (10,000 cells) treated with cisplatin. For the 3D model the cells grew on agarose coated plates. This data is an example of at least n=3.

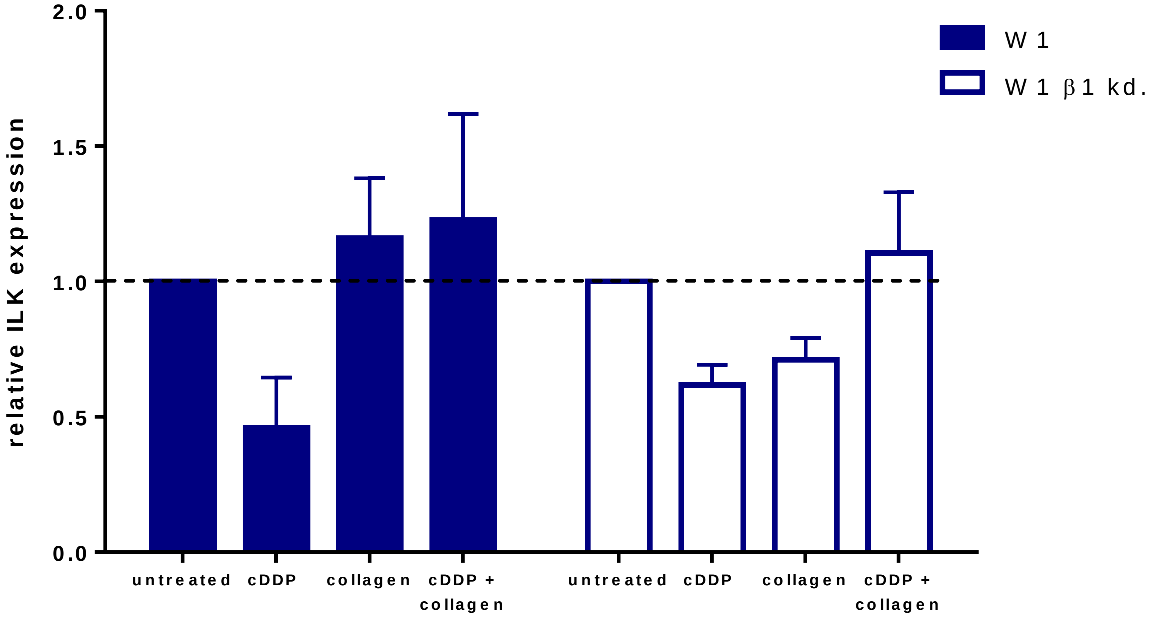


**Figure S2:** ILK expression profile in W1β1 kd cells compared to W1 wild type and the impact of COL1 and cisplatin treatment thereof.

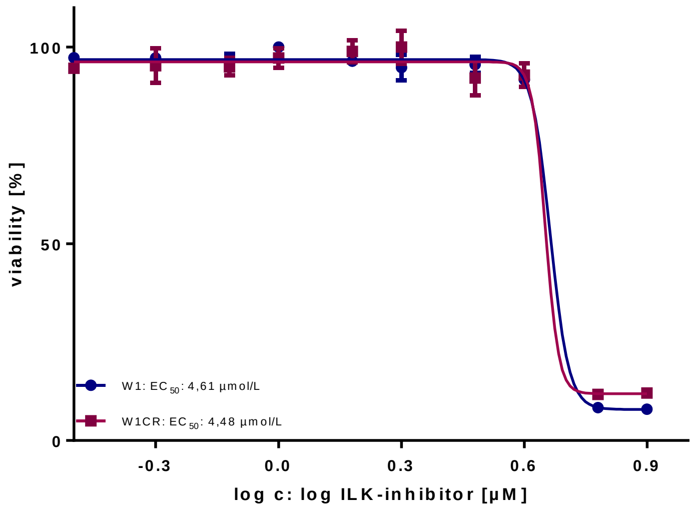


**Figure S3:** Concentration dependency of cell toxicity of the ILK inhibitor cdp22 in W1 and W1CR cells. The ILK inhibitor has no intrinsic cytotoxicity at the concentration used (1 µM) for interference with cell signaling. This data is an example of at least n=3.

**Table:**

| Cell number/well | treatment | W1 | | | W1CR | | | Rf $\left[\frac{W1CR}{W1}\right]$ |
|---|---|---|---|---|---|---|---|---|
| | | $pEC_{50}$ | | $EC_{50}$ | pEC50 | | $EC_{50}$ | |
| | | Mean | SE | [µmol/L] | Mean | SE | [µmol/L] | |
| 5,000 | cDDP | 5.92 | 0.06 | **1.19** | 5.17 | 0.11 | **6.71** | **5.63** |
| | cDDP + COL1 | 5.61 | 0.13 | **2.48** | 4.78 | 0.20 | **16.67** | **6.72** |
| 10,000 | cDDP | 5.76 | 0.17 | **1.75** | 4.94 | 0.15 | **11.42** | **6.54** |
| | cDDP + COL1 | 5.34 | 0.31 | **4.60** | 4.51 | 0.26 | **30.65** | **6.66** |

**Table S1**: $EC_{50}$ values of W1 and W1CR ovarian cancer cells with respect to cisplatin cytotoxicity and the impact of cell cultivation on COL1 surfaces. Data are means of at least n=3. (cDDP: cisplatin; COL1: collagen; SE: standard error; Rf: resistance factor; $EC_{50}$: half maximal effective concentration; $pEC_{50}$: negative decadal logarithm of the $EC_{50}$).

For the cell cytotoxicity assay, cells were seeded at two different cell densities (5,000 cells/well and 10,000 cells/well). To include potentially cell-cell contacts as an important factor in experiments with ECM.

Since the concentrations and the differences between the cell lines of ovarian carcinoma are very small, the $pEC_{50}$ value was used for statistical evaluation, such as averaging and calculation of the standard error, which presents itself as the decadal logarithm of the $EC_{50}$. The averaged $EC_{50}$ could thus be recovered from the statistically considered $pEC_{50}$. Thus, the resistant cells with cisplatin have an $EC_{50}$ higher by factor 6 (5.63). However, these are mean values from several experiments. While Figure 1A shows a single representative experiment.

Example: for the 5,000 W1 cells a mean $EC_{50}$ value of 1,19 µmol/L was obtained after cisplatin treatment. While the resistant W1CR cells have an $EC_{50}$ mean of 6,71 µmol/L after the same treatment.
