# Supplementary figures and images for: The Impact of Integrin-Mediated Matrix Adhesion on Cisplatin Resistance of W1 Ovarian Cancer Cells
Source: Biomolecules. 2019 Nov 26;9(12):788. doi: 10.3390/biom9120788 (PMC6995566; doi:10.3390/biom9120788)

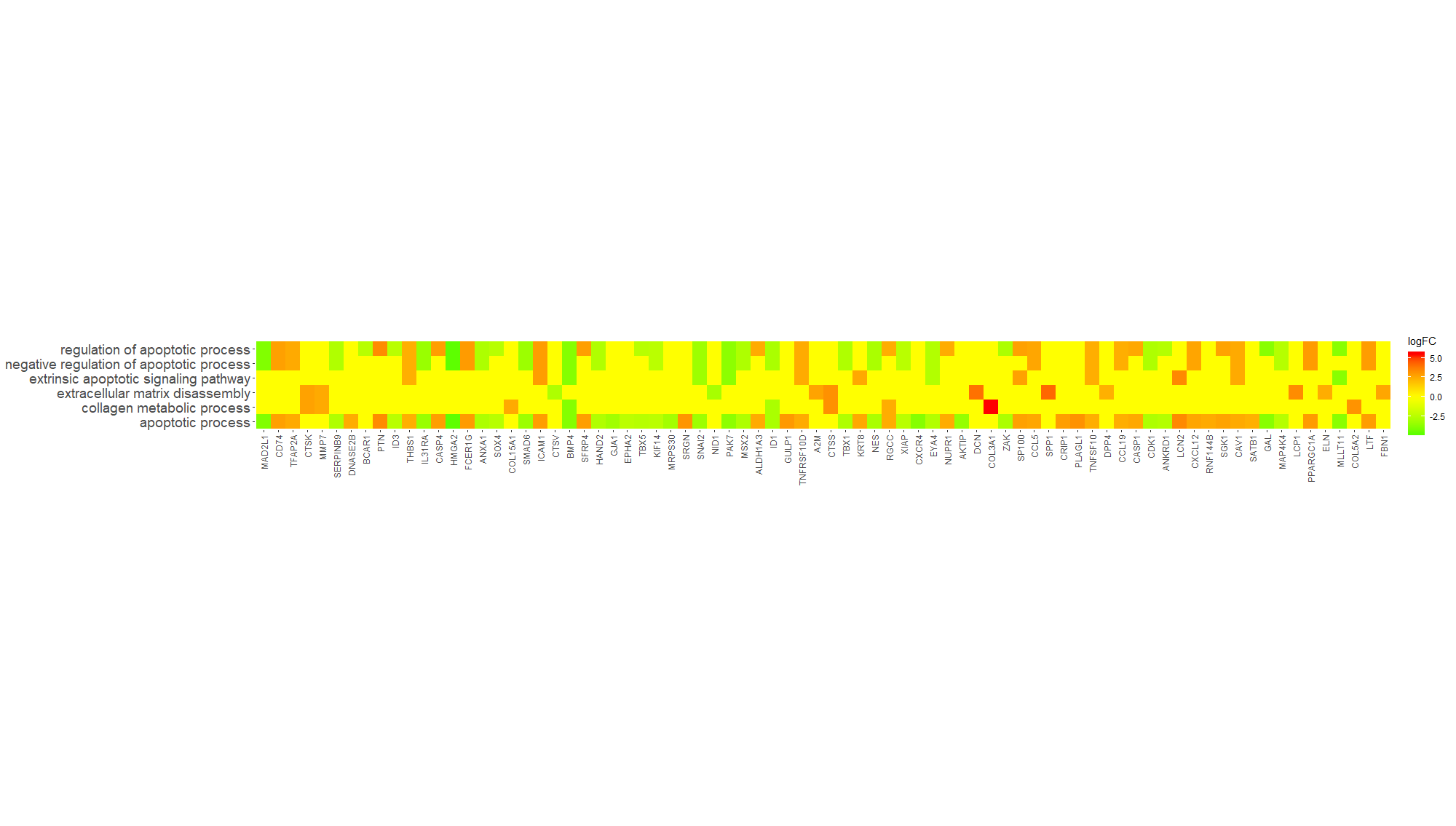

Supplement: Supplementary file 1 [file biomolecules-09-00788-s001.zip › HM_related_To_Fig1f_W1CR_vs_W1.tiff]

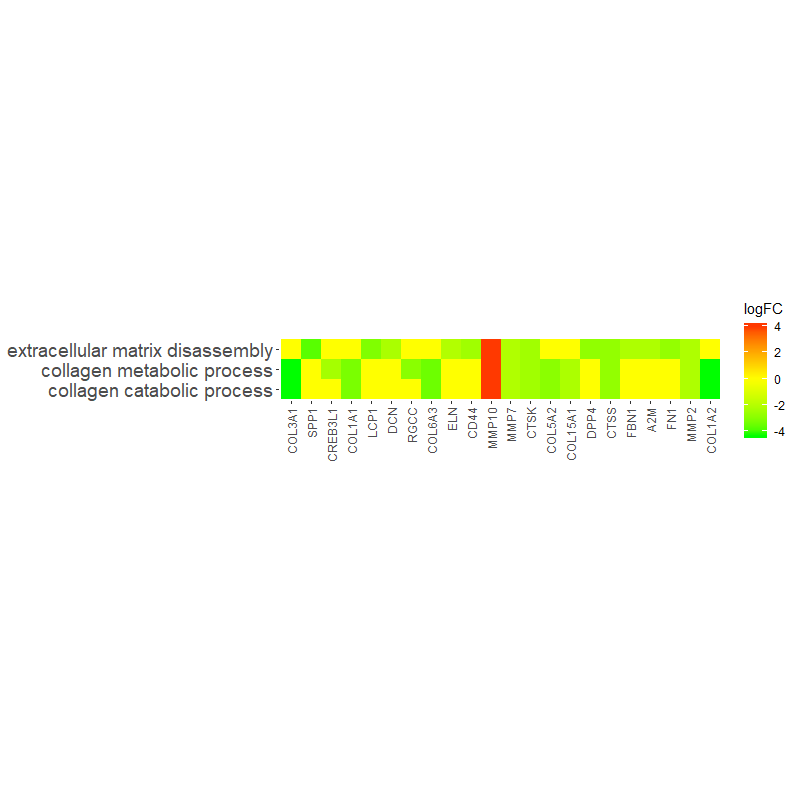

Supplement: Supplementary file 1 [file biomolecules-09-00788-s001.zip › HM_related_to_Fig3d_W1CRCollagen_vs_W1CR.tiff]
